# Obstetric, foetal and neonatal outcomes in adolescent pregnancy in sub-Saharan Africa: Systematic review and meta-analysis protocol

**DOI:** 10.1371/journal.pone.0323099

**Published:** 2025-05-09

**Authors:** Anthony Danso-Appiah, Eric Behene, Joan Naadu Hazel, Shirley Abanga

**Affiliations:** 1 Centre for Evidence Synthesis and Policy, University of Ghana, Accra, Ghana; 2 Department of Epidemiology and Disease Control, School of Public Health, University of Ghana, Accra, Ghana; 3 Africa Communities of Evidence Synthesis and Translation (ACEST), Accra, Ghana; 4 Department of Biostatistics, School of Public Health, University of Ghana, Accra, Ghana; 5 Department of Health Policy, Planning and Management, University of Ghana, Legon, Ghana; Kasr Alainy Medical School, Cairo University, EGYPT

## Abstract

**Background:**

Teenage pregnancy remains an important public health challenge in sub-Saharan Africa (SSA), with significant adverse health outcomes for the mother, their foetuses and infants. This systematic review will assess the impact of teenage or adolescent pregnancy on maternal obstetric, foetal and infant health outcomes, with the aim to inform evidence-based implementation of context-sensitive interventions and policies across countries in SSA.

**Methods:**

This protocol has been informed by the Cochrane handbook and other published works and reported in line with the Preferred Reporting Items for Systematic Reviews and Meta-Analyses for protocols (PRISMA-P) guidelines. We will search CINAHL, PubMed, Google Scholar, Scopus, Web of Science, Cochrane Library and LILACS, from inception to April 30, 2025, without language restriction. The search terms include ‘teenage pregnancy’, ‘adolescent pregnancy’, ‘maternal outcomes’, ‘obstetric outcomes’, ‘maternal death’, ‘maternal mortality’, ‘foetal outcomes’, ‘infant outcomes’ and ‘APGAR score’, together with their synonyms or alternate words, American or British spelling, singular or plural forms of the search terms or concepts. The names of all the 48 countries in SSA will be included as search terms. We will also search HINARI, African Journals Online, conference proceedings, preprint repositories, database of thesis, Specialized Obstetrics and World Bank Open Knowledge Repositories, WHO, PATH, and UNICEF databases. We will review the reference lists of relevant papers, and where necessary, contact experts in the field for their knowledge about studies missed by our searches. The retrieved studies will be managed in Endnote version X9 and duplicates removed. The deduplicated studies will be exported to Rayyan for study selection. At least two reviewers will independently screen and select studies using pre-tested study selection flow chart developed from the pre-specified eligibility criteria, extract the data using pretested data extraction form, and assess risk of bias in the included studies using ROBINS-E for non-randomized studies of exposure effects. Any disagreements will be resolved through discussion among the reviewers. We will analyze continuous outcomes as mean difference (MD) for studies using the same scales with their standard deviation (SD) and for studies measuring outcomes on different scales, the standardized mean difference (SMD) will be used. Binary/dichotomous outcome data will be expressed as odds ratio (OR) or risk ratio (RR). All the effect measures will be presented with their 95% confidence intervals (CIs). Pooled proportions will be used to determine prevalence or incidence of specific maternal, foetal and infant health outcomes. Heterogeneity will be assessed graphically and quantitatively using the I^2^ statistic. Random-effects meta-analysis will be used to synthesize data from the included studies, expected to be heterogeneous and with varying effect sizes. Where data permits, we will conduct subgroup analysis on key factors such age, trimester, gestation at delivery etc. to address the sources of heterogeneity. Where possible, sensitivity analysis will be performed to test the robustness of the pooled estimates around outlier variables and key risk of bias domains. The Grading of Recommendations Assessment Development and Evaluation (GRADE) approach will be used to assess the overall certainty of the evidence generated.

**Expected outcomes:**

This systematic review will inspire countries in Sub-Saharan Africa to implement innovative and context-sensitive programs that will improve the health and outcomes of adolescent pregnancy. An important step toward achieving this is to collate all empirical data through robust systematic review and meta-analysis to distill evidence at the highest level possible. Being the first review to rigorously identify and assess teenage pregnancy-related obstetric, foetal, and neonatal outcomes through the antenatal, labour to post-delivery (post-partum) periods within SSA, the review’s key findings will provide important evidence that will support efforts towards improving the wellbeing of teenage mothers and their newborns. Additionally, in countries across SSA where adolescent pregnancy rates are high and healthcare systems are constrained, this study may highlight important knowledge gaps that can be crucial in directing future research and resource allocation, for example, identifying the subgroup that will benefit from the limited resources. Also, the accumulated data to be pooled from these countries can shed light on the key factors influencing pregnancy outcomes and the trimesters during which these young mothers and their foetuses are most at risk and vulnerable. Such knowledge can help inform future policies and clinical practice.

**Protocol registration:**

This systematic review protocol has been registered in PROSPERO [CRD42024575044].

## Background

Adolescents or teenage pregnancy, defined as pregnancies from the ages of 10–19 years, is one of the most significant public health problems [1,2], even though most countries have made progress in reducing this public health threat [3,4]. Globally, rate of births by adolescent girls decreased from 64.5 births per 1000 women in 2000 to 41.3 in 2023 [5,6]. Despite regional decreases, sub-Saharan Africa (SSA) continues to have the highest rates worldwide, with 97.9 births per 1000 women in 2023 [7,8]. A multi country level analysis showed significant differences in teenage pregnancy rate across SSA, ranging from 75.6% in Chad, 44.3% in Congo, 36.5% in Rwanda, 29% in Malawi, 29% in Zambia, 3–23% in Ethiopia to 18% in Kenya [2,9,10], with up to three times as many teenage pregnancies in rural than in urban areas [11–13].

Several interrelated factors, including poverty [14,15], restricted educational opportunities [16,17], early marriage [18,19], female financial dependency on men [20,21], limited availability and access to sexual and reproductive health services [22,23], contribute to this high teenage pregnancy rate. These cultural and social factors not only increase the incidence of teenage pregnancies, but also the possibility of adverse outcomes [13,24,25]. Approximately, 3.9 million girls aged 15–19 years undergo unsafe abortions annually [10,26], which raises the risk of maternal mortality [26,27] as well as other acute and chronic pregnancy-related problems [28,29].

Pregnancy and delivery pose serious dangers for teenage mothers in SSA [2,4] unfavorable obstetric outcomes such as anemia [30,31], pre-eclampsia [32,33] and eclampsia [32,34] are common in teenage pregnancies [4]. Pregnancies among teenage girls are also associated with increased risk of premature labour [35,36], postpartum haemorrhage [37,38] and the need for blood transfusions [39,40]. Pregnant teenagers are more likely to experience fistulas [41], major perineal lacerations or episiotomy during childbirth [42,43], increased risk of maternal mortality [44,45] and postpartum depression [46,47] than adult pregnant women [40,48]. These health risks also extend to their foetuses and neonates [40,49].

The foetuses and infants of teenage mothers are at high risk of being stillborn [50,51], having low birth weight [49,50,52], being born preterm [50,53], having congenital malformations [54,55], experiencing low APGAR (Appearance, Pulse, Grimace, Activity, and Respiration) scores [56,57], being small for gestational age [49,58] and have increased tendencies to be admitted to the Neonatal Intensive Care Unit (NICU) [1,3,4,49,59]. Additionally, malnutrition and general health care pose serious challenges among adolescents which put their infants at a higher risk of sepsis and deaths [5,6]. Long-term developmental and behavioral needs that can extend into adulthood have also been documented [3,7].

### Rationale for this review

The highest proportion of teenage pregnancy has been estimated to occur in SSA [8,10,60]. Despite effort by governments and non-governmental organizations (NGOs) towards achieving the sustainable development goal aiming to reduce maternal mortality to less than 70 per 100,000 births by 2030 [11,12], the trend for teenage pregnancy in SSA is on a sharp rise. Complicating the situation is the fact that the relationship between age and pregnancy outcomes is not linear but involves complex interplay between various factors such as lifestyle, socioeconomic and demographic factors, even preexisting health conditions [13,61].

Teenage pregnancy is intimately linked to the sustainable development goal 3 (SDG 3) ─ Good Health and Well-Being, aimed at improving health and reducing morbidity and mortality of mothers [62,63] thereby preventing deaths of newborn and children under 5 [9,63]. The high number of teenage pregnancies in SSA [64–66], where health systems are frequently underfunded and constrained [67,68], makes it much more difficult to achieve SDG 3 targets. Meeting these targets will require tackling the high percentage of adolescent pregnancies, which greatly increases deaths among these vulnerable young mothers and their foetuses or newborns [2,9]. Even more, teenage pregnancy frequently results in high school dropout [69,70], complicating the situation further and widening the equitable education gap between girls and boys [69], which greatly impacts SDG 4─ Quality Education. Tackling teenage pregnancy and its associated consequences is essential in achieving the respective SDGs.

Preliminary searches conducted by our review team did not find any systematic review focusing on this topic in the sub-Saharan African context; only one systematic review exists but it investigated complications in childbearing young girls below 18 years [71]. Apart from being somehow outdated (synthesized data from 2005 to 2017) the study did not consider all girls within the teenage bracket which could potentially underestimate adverse pregnancy outcomes in this group. In contrast, our systematic review aims to determine the magnitude of adverse maternal pregnancy and foetal/neonatal outcomes during the antenatal (categorized into 1^st^, 2^nd^ and 3^rd^ trimesters), labour/delivery and postpartum periods and make recommendations for strategies aimed to improve maternal and child health outcomes in SSA. It seeks to answer the main question “What are the obstetric, foetal and neonatal outcomes in teenage and adolescent pregnancies in SSA? The specific questions are, 1) What is the proportion of teenage pregnancy in SSA? 2) What are the obstetric and foetal outcomes of teenage pregnancy in the antenatal period? 3) What are the obstetric and foetal adverse outcomes of teenage pregnancy during labour and delivery? and 4) What are the outcomes of teenage pregnancy during the post-partum period up to 28 days (neonatal period)? Objectively, this review seeks to: 1) determine the proportion of teenage pregnancy in SSA, 2) identify obstetric and foetal outcomes in teenage pregnancies during the antenatal period, 3) identify obstetric and foetal outcomes of teenage mothers during labour and delivery, and 4) determine obstetric and neonatal outcomes of teenage pregnancy during the postpartum period up to 28 days.

### Definitions of outcomes and key concepts/terms

- **Adolescent (or teenage pregnancy)**─ is pregnancy in a female under 20 years of age. It is a leading cause of death for girls aged 15–19 years, and babies of adolescent/teenage mothers are at higher risk of low birth weight, preterm birth, and severe neonatal conditions
- **Anaemia**─ where the blood has fewer red blood cells or lower haemoglobin levels than normal, leading to reduced oxygen-carrying capacity. During pregnancy, the body’s blood volume increases significantly, which can dilute the concentration of red blood cells, potentially leading to anaemia which is measured by haemoglobin (Hb) level. For the mother, it increases the risk of premature birth, low birth weight baby, postpartum depression, and blood loss during delivery and for the baby, risk of low birth weight, premature birth, and developmental problems
- **Antepartum**─ also known as the prenatal or antenatal period, refers to the time before childbirth, encompassing the entire period of pregnancy from conception to the onset of labour
- **Antepartum haemorrhage**─ bleeding from or into the genital tract, occurring from 24 + 0 weeks of pregnancy and prior to birth of the baby
- **APGAR scores**─ is a standardized, quick assessment to determine a newborn’s immediate health status, conducted at 1 and 5 minutes after birth, evaluating five areas: heart rate, breathing, muscle tone, reflexes, and skin color, each scored from 0 to 2
- **Birth asphyxia**─ is a condition in which a baby does not receive enough oxygen before, during, or directly after birth, a condition arising when the body is deprived of oxygen, causing unconsciousness or death
- **Caesarean section (C-section)**─ is a surgical procedure where a baby is delivered through an incision in the mother’s abdomen and uterus, typically performed when vaginal birth is deemed too risky for the mother or baby
- **Congenital anomalies**─ also known as birth defects, are structural or functional defects present at birth, arising from prenatal developmental issues and potentially impacting various body parts or systems. They can result from genetic factors, exposure to certain substances during pregnancy, infections, or a combination of factors
- **Difficult labour**─ also known as prolonged or obstructed labour, encompasses a slow or difficult progression of labour as a result of various problems such as slow dilation of the cervix, failure of the foetus to descend properly, where the baby’s head is too large to pass through the birth canal or the birth canal (cephalopelvic disproportion)
- **Eclampsia**─ severe pregnancy complication characterized by the onset of seizures in patients with uncontrolled pre-eclampsia
- **Failure to thrive**─ also known as growth faltering, is a condition where a child’s physical growth and development are significantly below expected levels, often due to inadequate nutrition, and is identified by weight or height below the 3rd or 5th percentile on growth charts
- **Foetal distress**─ also known as non-reassuring foetal status, refers to signs indicating a foetus is not receiving enough oxygen, and can be detected through abnormal foetal heart rate patterns, decreased movement, or meconium-stained amniotic fluid
- **Foetal growth restriction**─ also known as intrauterine growth restriction, occurs when the foetus fails to grow at the expected rate during pregnancy, resulting in a baby smaller than expected for its gestational age
- **Gestational diabetes**─ also called pregnancy-induced diabetes, is a condition where a woman develops high blood sugar (glucose) during pregnancy, usually disappearing after childbirth
- **Haemorrhage**─ vaginal bleeding during pregnancy, known as haemorrhage, can occur for various reasons, from common and benign causes like implantation bleeding to more serious issues like ectopic pregnancy or miscarriage
- **Hyperemesis gravidarum**─ is a severe and persistent nausea (an urge to vomit) and vomiting during pregnancy
- **Infant**─ a baby or very young child, normally from birth to around one year of age
- **Low birthweight**─ weight at birth of less than 2,500 grams, is a significant public health concern associated with increased ill-health and deaths among infants
- **Maternal depression**─ encompasses a range of depressive conditions affecting mothers during pregnancy (prenatal) and after childbirth (postpartum), including the “baby blues” and postpartum depression, which can impact a mother’s ability to bond with and care for her baby
- **Maternal anxiety**─ a feeling of fear, dread, and uneasiness, is experienced during pregnancy and can impact both the mother’s well-being and the developing foetus, potentially leading to issues in brain development and child behavior
- **Maternal deaths (mortality)**─ is defined as the death of a woman during pregnancy or within 42 days of termination of pregnancy, irrespective of the duration and site of the pregnancy, from any cause related to or aggravated by the pregnancy or its management, but not from accidental or incidental causes
- **Maternal sepsis**─ is a life-threatening condition that occurs when there is an infection during pregnancy, childbirth, post-abortion, or postpartum period. May lead to organ dysfunction, potentially leading to death or disability
- **Miscarriage**─ the spontaneous loss of pregnancy from conception to 24 weeks (28 weeks for many LMICs) of gestation (i.e., before the foetus reaches viability)
- **Mortality**─ another term for death. Mortality rate refers to the number of deaths that have occurred due to a specific illness or condition and is often expressed as the number of deaths due to an illness divided by the total population at that time
- **Neonatal sepsis**─ a serious bloodstream infection in infants under 28 days, often caused by bacteria and can be divided into early-onset (within 72 hours) and late-onset (after 72 hours) and is a leading cause of neonatal morbidity and mortality, especially in developing countries
- **Neonate**─ a child under 28 days of age. The child is at highest risk of dying during this period of life
- **Obstetrics**─ is the field of study concentrated on pregnancy, childbirth and the postpartum period
- **Obstetric fistula**─ is a serious, but preventable, childbirth injury characterized by an abnormal opening between the birth canal and bladder or rectum, leading to incontinence and significant health, social, and economic consequences for women
- **Obstructed labour**─ is a serious obstetric complication where the foetus fails to descend through the birth canal despite strong uterine contractions, often due to a mismatch between fetal size and the mother’s pelvis
- **Postpartum period**─ also known as the puerperium refers to the weeks following childbirth when the body undergoes physiological changes to return to a non-pregnant state, typically lasting 6–8 weeks but can be longer
- **Pre-eclampsia**─ also known as pregnancy-induced hypertension, is characterized by high blood pressure typically occurring after 20 weeks of pregnancy, and can lead to serious complications including seizures if left untreated
- **Premature rupture of membrane**─ occurs when the amniotic sac surrounding the baby ruptures before labour begins, and before 37 weeks of pregnancy
- **Prenatal period**─ also known as gestation, encompasses the time from conception to birth, typically lasting around 40 weeks, and is divided into three stages: first, second and third trimesters
- **Preterm (premature) labour**─ occurs when labour starts before 37 weeks of pregnancy, potentially leading to a preterm birth, which can cause serious health problems for the baby
- **Preterm (premature) birth**─ a baby born before 37 weeks of pregnancy, which is considered early and can lead to health problems for the baby. Preterm birth can occur spontaneously, meaning the labour starts on its own, or it can be induced where a doctor decides to induce labour or perform a Caesarean section due to health problems of the mother or baby
- **Postpartum depression**─ a mood disorder, is a type of depression that can occur during pregnancy or after childbirth, characterized by intense feelings of sadness, anxiety, or despair that can interfere with daily tasks
- **Postpartum haemorrhage**─ heavy bleeding after childbirth, is a serious condition that can be life-threatening if not treated quickly. It’s defined as losing more than 500 ml of blood after a vaginal birth or 1000 ml after a caesarean section. PPH is excessive bleeding after giving birth, which can occur within 24 hours of delivery (primary PPH) or later (secondary PPH)
- **Puerperal psychosis**─ also known as postpartum psychosis, is a severe mental illness that can occur shortly after childbirth, characterized by a sudden onset of psychotic symptoms like delusions, hallucinations, and confusion, requiring immediate psychiatric intervention
- **Small for gestational age**─ refers to a foetus or newborn who is smaller than what is expected for their gestational age, typically defined as a birth weight below the 10^th^ percentile (i.e., the foetus or newborn is smaller than expected for their gestational age). Small for gestational age can be caused by various factors, including genetic disorders, maternal health conditions, placental problems, or infections during pregnancy
- **Still birth**─ also known as foetal demise, is the death of a foetus after 20 weeks (or 28 weeks of viability in many LMICs) of pregnancy, but before birth, meaning the baby is born without signs of life
- **Uterine rupture**─ is a serious obstetric emergency where the uterus tears open, allowing the foetus, amniotic fluid, placenta to enter the abdominal cavity

## Methods

This systematic review protocol has been prepared in line with guidance specified in the Cochrane Handbook [72] and followed the methods reported in published systematic reviews [73–82]. We also followed the Preferred Reporting Items for Systematic Reviews and Meta-Analyses- extension for protocols (PRISMA-P) guidelines [83] (S1 Table). We will conduct comprehensive database searches as specified in this protocol and employ rigorous methods combined with best practices for the preparation of the full systematic review which will be reported following the PRISMA guidelines [84]. The study retrieval and selection process will be reported using the PRISMA Flow Diagram (S1 Fig).

### Criteria for considering studies for inclusion in this systematic review

#### Types of studies.

Guided by earlier published works [73–75], observational studies including cohort, case-control and cross-sectional studies that assessed maternal, foetal and child health outcomes among teenage mothers in SSA countries will be eligible for inclusion in this systematic review. Only studies that reported a well-defined sample or representative sub-sample of the source population will be eligible for inclusion. Though studies that assessed intervention effectiveness will not be the focus of this systematic review, trials that reported baseline data to allow the calculation of proportion of teenage pregnancy outcomes, will be eligible for inclusion. Reviews, commentaries, opinions, case-studies and case-series will not be eligible for inclusion. However, if a potentially relevant primary study is identified in the reviews, commentaries, opinions, case-studies and case-series, it will be retrieved and assessed for eligibility. For multi-country study with global focus, we will include only the studies coming from SSA for inclusion. Where data from multi-country studies have been pooled and there is no way we could disaggregate the data, such a study will not be eligible for inclusion. Also, if a study has lumped data on adults and teenage mothers, we will attempt to disaggregate the data and include only the data for teenage mothers. However, if disaggregation of the data is not possible, we will exclude it from the analysis.

#### Participants.

Adolescents or teenage girls aged 10 to and 19 years living in a country in SSA who became pregnant will be eligible for inclusion in this systematic review. The criteria for the identification of pregnancy should be stated. Any adolescent or teenage woman whose pregnancy could not be confirmed will be excluded from the study.

#### Intervention.

The focus of this systematic review is not intervention effectiveness.

#### Comparator.

Adult pregnant women living in SSA. When data permit, we will conduct subgroup comparisons, for example, teenage mothers from rural versus urban areas, older teenage versus very young teenage pregnant women.

### Outcomes

#### Primary outcomes.

- Deaths associated with adolescent/teenage pregnancy
- Perinatal deaths (stillbirth + death within a week of delivery)
- Admission to Neonatal Intensive Care Unit

#### Secondary outcomes.

***Antenatal (we will categorize into 1***^***st***^***, 2***^***nd***^ ***and 3***^***rd***^ ***trimesters where necessary***)

- *Maternal outcomes*
- Proportion of teenagers becoming pregnant in countries in SSA
- Age at first pregnancy
- Proportion with anaemia for both mother and baby (measured by Hb levels)
- Pregnancy-induced hypertension (pre-eclampsia)
- Eclampsia
- Gestational diabetes
- Hyperemesis gravidarum
- Antepartum haemorrhage
- Miscarriage
- Induced abortion
- Maternal deaths
- Depression
- Anxiety
- *Foetal outcomes*
- Small for gestational age
- Congenital anomalies
- Foetal growth restriction
- Stillbirth

***Labour/delivery***

- *Maternal outcomes*
- Maternal deaths
- Preterm labour
- Haemorrhage
- Difficult labour (including prolonged and obstructed labour, etc.)
- Premature rupture of membrane
- Caesarean section
- Uterine rupture
- Obstetric fistula
- *Foetal outcomes*
- Asphyxia
- Foetal distress
- Admission to Neonatal Intensive Care Unit

***Postpartum***

- *Maternal outcomes*
- Postpartum haemorrhage
- Postpartum depression
- Puerperal psychosis
- Maternal sepsis
- *Neonatal outcomes (up to 28 days post-delivery)*
- Preterm birth/delivery
- APGAR scores
- Neonatal sepsis (up to 28 days after delivery)
- Stillbirth
- Failure to thrive
- Low birthweight
- Congenital anomalies (including cerebral palsy, down syndrome etc.)

### Search for studies

We will retrieve all studies (published and unpublished) and assess their eligibility for inclusion. The following databases will be searched: PubMed, CINAHL, LILACS, Google Scholar, Scopus, Web of Science, Cochrane Library, LILACS, from inception to April 30, 2025, without language restriction. The search terms and concepts include ‘teenage pregnancy’, ‘adolescent pregnancy’, ‘maternal outcomes’, ‘obstetric outcomes’, ‘foetal outcomes’, ‘neonatal outcomes’, ‘maternal death’, ‘maternal mortality’, ‘infant outcomes’ and ‘APGAR score’, together with their synonyms or alternate words, American or British spelling, singular or plural of the search terms or concepts. The names of all the 48 countries in ‘sub-Saharan Africa will be included as search terms (see Table 1 for the search strategy developed for PubMed which will be adapted for the other databases). Additional database and non-database sources such as HINARI, African Journals Online, WHO, PATH, and UNICEF databases, conference proceedings, and database of thesis/dissertation, as well as preprint and the World Bank Open Knowledge Repositories will be searched for more studies. We will go through the reference lists of relevant articles and where necessary, contact field experts for their knowledge about any study missed by our searches.

**Table 1:** Search strategy for PubMed (to be tailored to other databases).

Search	Querry	Results
#1	(“Teenage pregnancy”[Title/Abstract]) OR (“Adolescent pregnancy”[Title/Abstract])	
#2	((((((((((((((((((((((((((((((((((((((“Maternal outcomes “[Title/Abstract]) OR (“Maternal anaemia “[Title/Abstract])) OR (“Maternal anaemia “[Title/Abstract])) OR (haemorrhage[Title/Abstract])) OR (“Antepartum haemorrhage “[Title/Abstract])) OR (Antepartum[Title/Abstract])) OR (Haemorrhage[Title/Abstract])) OR (Hypertension[Title/Abstract])) OR (Preeclampsia[Title/Abstract])) OR (Eclampsia[Title/Abstract])) OR (“Hyperemesis gravidarum “[Title/Abstract])) OR (Miscarriage[Title/Abstract])) OR (pregnancy-induced[Title/Abstract])) OR (“HELLP syndrome”[Title/Abstract])) OR (“Maternal mortality”[Title/Abstract])) OR (“Anxiety disorder”[Title/Abstract])) OR (“Maternal death”[Title/Abstract])) OR (“Maternal mortality”[Title/Abstract])) OR (depression[Title/Abstract])) OR (anxiety[Title/Abstract])) OR (“postpartum depression “[Title/Abstract])) OR (“obstetric fistula “[Title/Abstract])) OR (“obstetric fistulas “[Title/Abstract])) OR (“postpartum haemorrhage “[Title/Abstract])) OR (“postpartum haemorrhage “[Title/Abstract])) OR (“Preterm labour”[Title/Abstract])) OR (“Preterm labour “[Title/Abstract])) OR (“Prolonged labour “[Title/Abstract])) OR (“Prolonged labour “[Title/Abstract])) OR (“Difficult labour “[Title/Abstract]))) OR (“Premature rupture of membrane”[Title/Abstract])) OR (“Caesarean section”[Title/Abstract])) OR (“Caeserean section “[Title/Abstract])) OR (“Uterine rupture “[Title/Abstract])) OR (“Obstructed labour “[Title/Abstract])) OR (“Obstructed labour “[Title/Abstract])) OR (“Puerperal psychosis “[Title/Abstract])) OR (“Maternal sepsis”[Title/Abstract])	
#3	(#1) AND (#2)	
# 4	((((((((((((“Foetal outcomes “[Title/Abstract]) OR (“foetal outcomes “[Title/Abstract])) OR (“small for gestational age “[Title/Abstract])) OR (“congenital defects “[Title/Abstract])) OR (“congenital anomaly “[Title/Abstract])) OR (“sepsis”[Title/Abstract])) OR (“failure to thrive “[Title/Abstract]))) OR (“Foetal growth retardation “[Title/Abstract])) OR (“Foetal growth restriction “[Title/Abstract])) OR (asphyxia[Title/Abstract])) OR (“Foetal distress”[Title/Abstract])) OR (“Foetal distress “[Title/Abstract])	
#5	(#3) AND (#4)	
# 6	(((((((((((((((((“Neonatal outcomes “[Title/Abstract]) OR (“Child outcomes “[Title/Abstract])) OR (“down syndrome “[Title/Abstract])) OR (“cerebral palsy “[Title/Abstract])) OR (“infant mortality “[Title/Abstract])) OR (“infant death “[Title/Abstract])) OR (“Neonatal sepsis “[Title/Abstract])) OR (infection[Title/Abstract])) OR (“APGAR score “[Title/Abstract])) OR (“cerebral palsy “[Title/Abstract])) OR (wasting[Title/Abstract])) OR (“developmental disorders “[Title/Abstract])) OR (Stillbirth[Title/Abstract])) OR (“preterm birth “[Title/Abstract])) OR (prematurity[Title/Abstract])) OR (“low birth weight “[Title/Abstract])) OR (“congenital anomalies “[Title/Abstract])) OR (“Failure to thrive”[Title/Abstract])	
# 7	(((((((((((((((((((((((((((((((((((((((((((((((((“Democratic Republic of Congo”[Title/Abstract]) OR (“Republic of Congo”[Title/Abstract])) OR (“Central African Republic”[Title/Abstract])) OR (Rwanda[Title/Abstract])) OR (Burundi[Title/Abstract])) OR (Sudan[Title/Abstract])) OR (Kenya[Title/Abstract])) OR (Tanzania[Title/Abstract])) OR (Uganda[Title/Abstract])) OR (Djibouti[Title/Abstract])) OR (Eritrea[Title/Abstract])) OR (Ethiopia[Title/Abstract])) OR (Somalia[Title/Abstract])) OR (Angola[Title/Abstract])) OR (Botswana[Title/Abstract])) OR (Lesotho[Title/Abstract])) OR (Malawi[Title/Abstract])) OR (Mozambique[Title/Abstract])) OR (Namibia[Title/Abstract])) OR (“South Africa”[Title/Abstract])) OR (Swaziland[Title/Abstract])) OR (Zambia[Title/Abstract])) OR (Zimbabwe[Title/Abstract])) OR (Benin[Title/Abstract])) OR (“Burkina Faso”[Title/Abstract])) OR (Cameroon[Title/Abstract])) OR (Chad[Title/Abstract])) OR (“Côte d’Ivoire”[Title/Abstract])) OR (“Equatorial Guinea”[Title/Abstract])) OR (Gabon[Title/Abstract])) OR (Gambia[Title/Abstract])) OR (Ghana[Title/Abstract])) OR (Guinea[Title/Abstract])) OR (Guinea-Bissau[Title/Abstract])) OR (Liberia[Title/Abstract])) OR (Mali[Title/Abstract])) OR (Mauritania[Title/Abstract])) OR (Niger[Title/Abstract])) OR (Nigeria[Title/Abstract])) OR (Senegal[Title/Abstract])) OR (“Sierra Leone”[Title/Abstract])) OR (Togo[Title/Abstract])) OR (“Cape Verde”[Title/Abstract])) OR (Comoros[Title/Abstract])) OR (Madagascar[Title/Abstract])) OR (Mauritius[Title/Abstract])) OR (“São Tomé and Príncipe”[Title/Abstract])) OR (Seychelles[Title/Abstract])) OR (“Sub-Saharan Africa”[Title/Abstract])) OR (SSA[Title/Abstract])	
# 8	(# 5) AND (#6) AND (#7)	

### Managing the search results and selecting studies

**Fig 1:**
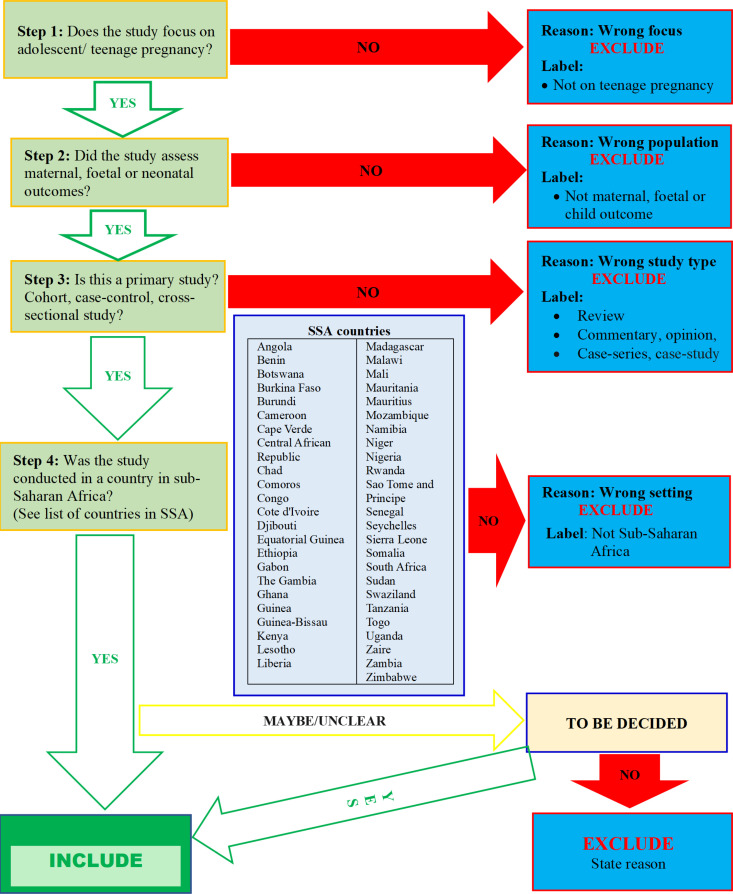
Study selection flowchart developed from the pre-specified eligibility criteria.

All articles retrieved from the various electronic databases and other sources (non-electronic database sources) will be uploaded into Endnote version X9 where duplicates will be removed. The deduplicated articles will be exported to Rayyan (https://rayyan.qcri.org/welcome), where study selection will be conducted using pre-tested study selection flow chart (Fig 1). At least two reviewers will independently screen and select studies.

First, titles and abstracts will be screened to remove completely irrelevant studies. The full text of articles that potentially meet the inclusion criteria will be sought and screened for inclusion. At this stage, any study that did not meet the inclusion criteria will be excluded and the reason for exclusion will be provided. The flow of studies through the selection process will be presented using the PRISMA flow diagram. Any discrepancies or disagreements between the reviewers will be resolved through discussion. When necessary, a third team member will intervene to mediate conflicts.

### Data extraction and management

At least two reviewers will extract data using pretested data extraction form (S2 Table) to collect information on the characteristics of the studies including year the study was conducted, year it was published, the country where the study was conducted, the publication language, study design and the number of participants involved (sample size). Sociodemographic characteristics of the participants such as age, level of education, occupation, setting and marital status will also be extracted. We will extract outcome data at the following levels: antenatal for teenage mothers (proportion of teenagers becoming pregnant in countries in SSA, pregnancy-induced hypertension (pre-eclampsia), eclampsia, gestational diabetes (pregnancy-induced diabetes), hyperemesis gravidarum (extreme, persistent nausea and vomiting during pregnancy), antepartum haemorrhage (APH) (bleeding occurring from 24 weeks of pregnancy and prior to delivery), miscarriage, anaemia (measured by Hb level), depression and anxiety, and foetal outcomes such as small for gestational age, congenital anomalies and foetal growth restriction; labour/delivery outcomes such as preterm labour, haemorrhage, difficult labour (including prolonged labour), premature rapture of membrane (PROM), caesarean section, uterine rupture, obstetric fistulas, obstructed labour and maternal deaths etc., and foetal outcomes such as asphyxia, foetal distress, etc.; and postpartum outcomes for the mother including postpartum haemorrhage (PPH), defined as heavy bleeding after delivery up to 12 weeks post-delivery, postpartum depression, puerperal psychosis, maternal sepsis etc., and for neonatal outcomes such as preterm birth/delivery, APGAR scores, neonatal sepsis, still birth, failure to thrive, low birthweight and congenital anomalies including cerebral palsy and down syndrome, etc. The extracted data will be cleaned and managed with Microsoft Excel where all transformations and conversion needed for the analysis will be done. Any discrepancies or disagreements in the data extracted will be resolved through discussion between the review authors.

### Risk of bias in the included studies (Quality assessment)

At least two reviewers will independently assess quality in the included studies for risk of bias using the Cochrane tool for non-randomized studies of exposure (Robbins- E) (Version 20 June 2023) (S3 Table). This tool is based on a series of signaling questions across seven risk of bias domains: 1) confounding, 2) selection of study participants, 3) measurement of exposure, 4) post-exposure intervention, 5) missing data, 6) measurement of outcome, and 7) selection of reported results. Responses to each of the signaling questions are ‘Yes’, ‘Probably Yes’, ‘Probably No’, ‘No’, and ‘No Information’. The risk of bias will be judged as ‘low’ for a domain with little or no concern about bias, ‘Some Concerns’ where there are some concerns about bias in a specific domain but with no certainty of an important risk of bias, ‘High risk’ for domains with some important bias concerns, and ‘very high risk of bias for studies with suspected serious bias. The results from the risk of bias assessment will be presented in a table with supporting statements from the primary studies. The risk of bias in prevalence studies will be assessed using Hoy et al. [85] quality assessment tool (S4 Table). This tool consists of 10 items that address four risk of bias domains: 1) representativeness of the study sample to the source population, 2) representativeness of the sampling frame to the target population, 3) reliability of the sampling, 4) likelihood of minimization of nonresponse bias, 5) data collected directly from the subjects as opposed to a proxy, 6) acceptable case definition, 7) validity and reliability of study instrument that measured the parameter of interest, 8) mode of data collection used, 9) appropriateness of the prevalence period for the parameter of interest, and 10) appropriateness of the numerator(s) and denominator(s) for the parameter of interest. The first four domains assess external validity and domains 5–10 assess internal validity. The risk of bias in each domain will be judged as “low”, “high” or “unclear”. The internal validity domains will be the basis for scoring the overall risk of bias in each of the included studies as “low-” or “high-” risk of bias. Any disagreements will be resolved through discussion between the review authors.

### Managing missing and incomplete data

For studies with incomplete or missing data, we will attempt to contact authors of the primary study and request the raw data or for them to provide additional or the missing data. We will not impute the missing data where the primary study authors are unable or refuse to provide the requested data or when they cannot be reached. In such cases, we will report the data as missing and discuss the potential impact and implications of the missing data on the review’s findings and conclusions in the discussion.

### Plan for data-, subgroup-, and sensitivity- analysis

We will use Stata Version 17 for the data analysis. Non-comparative studies will be combined to generate an overall (pooled) estimate (proportions, percentages etc.) which will be presented with their 95% confidence intervals (CIs). Binary outcome data (prevalence, incidence etc.) will be analyzed as odds ratio (OR) or risk ratio (RR), and continuous data as mean difference (MD) with standard deviation (SD); all estimates will be reported with 95% CIs. Mantel-Haenszel random-effects meta-analysis will be used to synthesize data from the included studies to generate pooled proportions, OR, RR or MD and displayed graphically as forest plots. Heterogeneity arising from variations in the study design, participants characteristics, outcomes and the instruments/scales used will be assessed quantitatively using the I-squared (I^2^) statistic. Subgroup analysis will be conducted to explore the potential impact of heterogeneity on the pooled effect estimates. The factors to be considered for subgroup analysis include study design, age at first pregnancy, diagnostic criteria, age, trimester (first, second, third), setting (rural or urban) and region where the study was conducted (East, West, Central and Southern Africa). Sensitivity analysis will be performed on outlier studies and key risk of bias domains to test the robustness of the pooled estimates obtained from the meta-analysis.

### Assessing certainty of evidence using GRADE

Informed by earlier published work [86], we will use the Grading of Recommendations Assessment, Development and Evaluation (GRADE) approach [87] to assess the overall quality or certainty of the evidence (how certain the authors are that the effect estimate represents the true effect) on the key outcomes provided by the included studies. At least two reviewers will assess the certainty of evidence (*quality* of evidence or the *confidence* in the effect estimates) from the following five domains: risk of bias, inconsistency, indirectness, imprecision, or publication bias. The certainty of evidence will be graded as high, moderate, low, or very low. The certainty of evidence can be rated down by one or two levels when there are serious or very serious concerns, respectively, in any of the domains [87,88]. The certainty of evidence will be graded upward for high-quality observational studies, although this is usually rarely done [88,89]. An overall rating of the certainty of the evidence for each outcome will be presented in a summary of findings table together with the study types, the number of studies and participants, and the relative and absolute effects for each outcome [89].

### Patient and public involvement

The review questions and outcome measures have been developed collaboratively with the relevant patients and public stakeholder involvement and informed by their priorities, experiences, and preferences. We followed the guidelines specified in the Guidance for Reporting Involvement of Patients and the Public (GRIPP2) checklist [90]. The review findings will be shared with the wider patient communities, who will also be involved in the dissemination of the results.

### Ethics and dissemination

Ethical approval is not required for a systematic review as it involves synthesizing data from previously published works that have already received ethical approval. The findings from this review will be submitted for peer-reviewed publication, presented at scientific conferences, and shared with stakeholders and policymakers.

## Discussion

Teenage pregnancy is a serious public health issue that affects maternal, foetal and newborn health and wellbeing in profound ways. The relationship between teenage pregnancy and unfavourable obstetric, foetal, and neonatal outcomes has been the subject of numerous studies, yielding different outcomes for various groups and environments. This systematic review is the very first to attempt to collate evidence, in a comprehensive manner, on obstetric, foetal and neonatal outcomes of teenage pregnancy in the antenatal, labour/delivery, and post-partum periods in SSA in order to estimate the magnitude adverse outcomes and risk to the health of expectant adolescents/teenagers and their babies. The review will be useful for shaping policies, clinical practice and future research. This review employs rigorous methods to address both external and internal validity issues that usually affect the reliability of systematic reviews findings and conclusions. External validity employs PICOS to concisely delineate the attributes of the participants, interventions, outcomes and study designs whereas the internal validity element addresses mainly the risk of bias (quality) domains. The review gathers evidence through a very comprehensive search strategy that aims to locate all potentially relevant studies from a variety of sources (database and non-database sources) and study design types, including cohort, case-control, and cross-sectional studies. It does this by using transparent and robust methods (as detailed in the methods) to distil evidence at the highest level possible, minimizing flaws and biases. It is anticipated that the review findings will provide policy makers, health providers and other stakeholders with evidence-based information and inspire countries to adopt innovative interventions to limit adverse outcomes of adolescent/teenage pregnancy.

### Study limitations

There could be a possible publication bias which could have an impact on the review and meta-analysis due to negative results regarding adolescent/teenage pregnancy adverse outcomes being underreported. The review may include research from various settings with distinct healthcare systems and cultural norms, which will limit generalizability of the findings due to geographic and socioeconomic disparities in the outcomes of teenage pregnancy. Comprehensive analysis may also be limited by incomplete reporting of relevant outcomes.

### Implications of the anticipated review findings

The strength of this review will be the pooling of data from different studies from different countries across SSA to determine obstetric, foetal and neonatal outcomes in adolescent pregnancy which is critical for shaping policies, clinical practice and research. This review is expected to draw attention to the need for stronger, adolescent-focused maternal healthcare services by highlighting the increased risks of obstetric, foetal, and neonatal outcomes as a result of teenage pregnancy. The results could also point to differences in health outcomes according to socioeconomic and regional characteristics, highlighting the need for equitable resource allocation and focused public health interventions. These findings may help shape health policies that support better access to contraception, comprehensive sexual education, and discourage actions that promote adolescent/teenage pregnancies. This review may also point out gaps in the current body of knowledge about teenage pregnancy and its associated maternal, foetal and neonatal outcomes, which could direct future research to address understudied topics and, in the end, improve the health of adolescent mothers and their offsprings. These results will be very important in achieving SDG 3, particularly with regards to lowering maternal and newborn deaths in sub-Saharan Africa.

## Supporting information

S1 Fig PRISMA flow diagram. (PDF)

S1 Table PRISMA-P guidelines. (PDF)

S2 Table Data Extraction form. (PDF)

S3 Table Robbins-E risk of bias assessment tool. (PDF)

S4 Table Risk of bias tool for prevalence studies. (PDF)
