# Supplementary material for: Obstetric, foetal and neonatal outcomes in adolescent pregnancy in sub-Saharan Africa: Systematic review and meta-analysis protocol
Source: PLoS One. 2025 May 9;20(5):e0323099. doi: 10.1371/journal.pone.0323099 (PMC12063885; doi:10.1371/journal.pone.0323099)
Supplement: S1 Fig — (PDF) [file pone.0323099.s001.pdf]

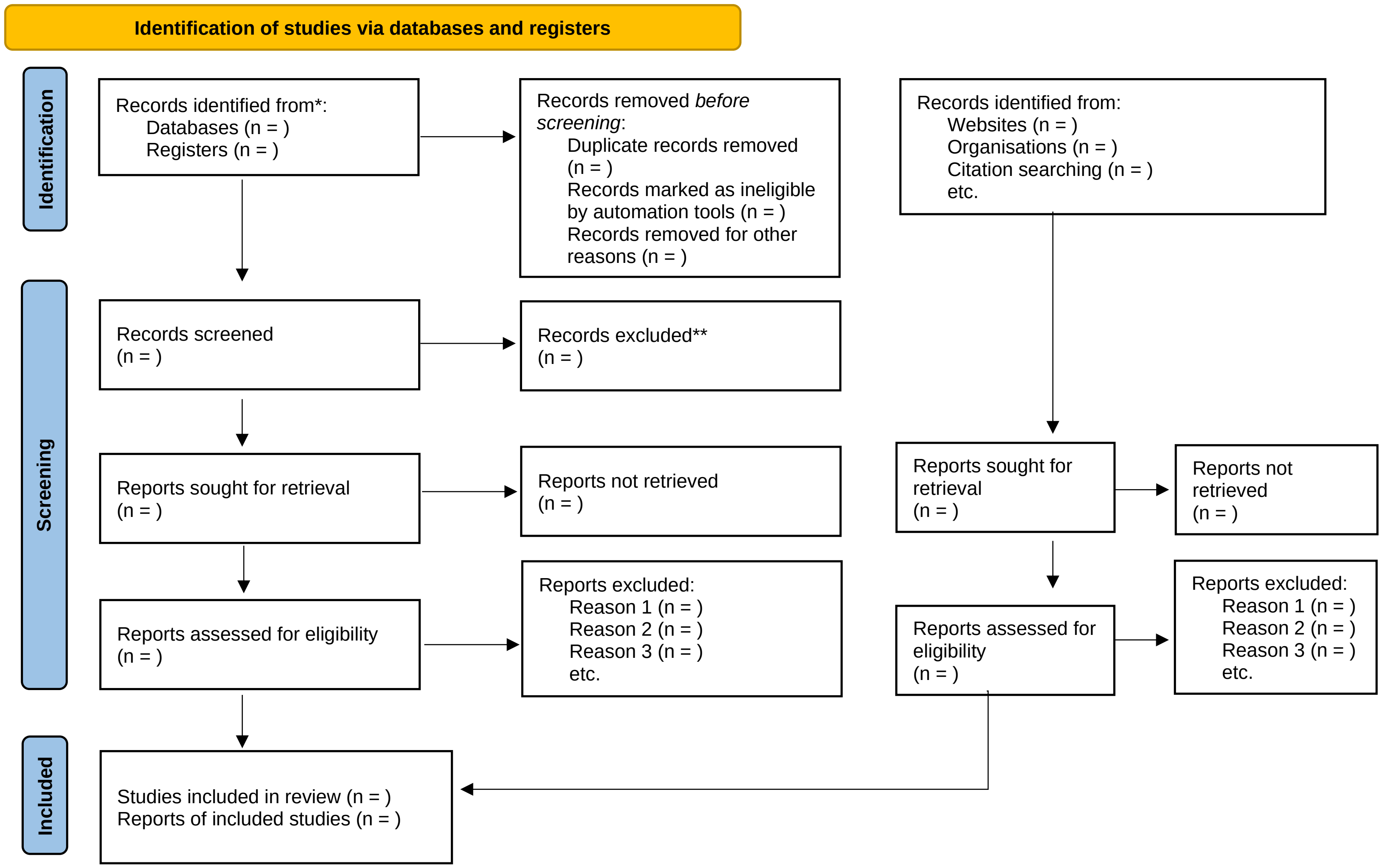

SUPLEMENTAL FILE 2 PRISMA-P 2020 Flow Diagram to show studies retrieved from electronic databases and other sources for inclusion and flow to the final stage with studies included in the systematic review
