# Supplementary material for: Obstetric, foetal and neonatal outcomes in adolescent pregnancy in sub-Saharan Africa: Systematic review and meta-analysis protocol
Source: PLoS One. 2025 May 9;20(5):e0323099. doi: 10.1371/journal.pone.0323099 (PMC12063885; doi:10.1371/journal.pone.0323099)
Supplement: S3 Table — (PDF) [file pone.0323099.s004.pdf]

Additional file 3: Robbins E Risk of Bias Assessment Tool

Domain 1: Risk of bias due to confounding

Domain 1, Variant (a): ***If N/PN to C5 or Y/PY to C6 or N/PN to C7*** *(only baseline confounding needs to be addressed)*

**Signalling questions**	**Response options**
1.1 Did the authors control for all the important confounding factors for which this was necessary?	Y / PY / WN (no, but uncontrolled confounding was probably not substantial) / SN (no, and uncontrolled confounding was probably substantial) / NI
1.2 **If Y/PY/WN to 1.1**: Were confounding factors that were controlled for (and for which control was necessary) measured validly and reliably by the variables available in this study?	NA / Y / PY / WN (no, but the extent of measurement error in confounding factors was probably not substantial) / SN (no, and the extent of measurement error in confounding factors was probably substantial) / NI
1.3 **If Y/PY/WN to 1.1**: Did the authors control for any variables after the start of the exposure period being studied that could have been affected by the exposure?	NA / Y / PY / PN / N / NI
1.4 Did the use of negative controls, or other considerations, suggest serious uncontrolled confounding?	Y / PY / PN / N
Risk of bias (due to confounding) in the estimated effect of exposure on the outcome	Low risk / Some concerns / High risk / Very high risk
What is the predicted direction of bias due to confounding?	(Towards benefit of (higher) exposure / Towards harm of (higher) exposure / Insufficient information available)
Is the risk of bias (due to confounding) sufficiently high, in the context of its likely direction and the magnitude of the estimated exposure effect, to threaten conclusions about whether the exposure has an important effect on the outcome?	Yes / No / Cannot tell

Y = Yes; PY = Probably yes; PN = Probably no; N = No; SY = Strong yes; WY = Weak yes; SN = Strong no; WN = Weak no; NA = Not applicable; NI = No information

Domain 2: Risk of bias arising from measurement of the exposure

Domain 2, Variant (a): ***If N/PN to C5*** *(exposure was measured at a single point in time)*

**Signalling questions**	**Response options**
Mismeasurement or misclassification of the exposure.	
2.1 Does the measured exposure well-characterize the exposure metric specified to be of interest in this study? [*This was specified in the answers to D2, D3 and D4*]	Y / PY / WN (no, to a small extent) / SN (no, to a large extent) / NI
2.2 Was the exposure likely to be measured with error, or misclassified?	SY (yes, probably a substantial amount) / WY (yes, but probably not a substantial amount) / PN / N / NI
Bias in the estimated effect of exposure arising from mismeasurement or misclassification of the exposure	
2.3 If SY/WY to 2.2: Could mismeasurement or misclassification of exposure have been differential (i.e. related to the outcome or risk of the outcome)?	NA / SY (yes, to a large extent) / WY (yes, to a small extent) / PN / N / NI
2.4 If SY/WY to 2.2 and N/PN/WY to 2.3: Is non-differential measurement error likely to bias the estimated effect of exposure on outcome?	NA / SY (yes, to a large extent) / WY (yes, to a small extent) / PN / N / NI
Risk of bias (arising from measurement of exposure) in the estimated effect of exposure on the outcome	Low risk / Some concerns / High risk / Very high risk
What is the predicted direction of bias arising from measurement of exposure?	Towards benefit of (higher) exposure / Towards harm of (higher) exposure / Towards null / Away from null / Insufficient information available
Is the risk of bias (arising from measurement of exposure) sufficiently high, in the context of its likely direction and the magnitude of the estimated exposure effect, to threaten conclusions about whether the exposure has an important effect on the outcome?	Yes / No / Cannot tell

Y = Yes; PY = Probably yes; PN = Probably no; N = No; SY = Strong yes; WY = Weak yes; SN = Strong no; WN = Weak no; NA = Not applicable; NI = No information

Domain 3: Risk of bias in selection of participants into the study (or into the analysis)

Signalling questions	Response options
3.1 Did follow-up begin at (or close to) the start of the exposure window for most participants? [*The exposure window is specified in D3*]	Y / PY / PN / N / NI
3.2 **If N/PN to 3.1**: Is the effect of exposure likely to be constant over the period of follow up analysed?	NA / Y / PY / PN / N / NI
3.3 Was selection of participants into the study (or into the analysis) based on participant characteristics observed after the start of the exposure window being studied? [*The exposure window is specified in D3*]	Y / PY / PN / N / NI
3.4 **If Y/PY to 3.3**: Were these characteristics likely to be influenced by exposure or a cause of exposure?	NA / Y / PY / PN / N / NI
3.5 **If Y/PY to 3.4**: Were these characteristics likely to be influenced by the outcome or a cause of the outcome?	NA / Y / PY / PN / N / NI
3.6 **If N/PN to 3.2 or Y/PY to 3.5**: Is it likely that the analysis corrected for all of the potential selection biases identified in A and B above?	NA / Y / PY / PN / N / NI
3.7 **If N/PN to 3.2 or Y/PY to 3.5**: Did sensitivity analyses demonstrate that the likely impact of the potential selection biases identified in A or B above was minimal?	NA / Y / PY / WN (no, there were no sensitivity analyses or there is evidence of some impact) / SN (no, there is evidence of substantial impact)
Risk of bias (due to selection of participants into the study) in the estimated effect of exposure on the outcome	Low risk / Some concerns / High risk / Very high risk
What is the predicted direction of bias<br><br>due to selection of participants into the study?	Towards benefit of (higher) exposure / Towards harm of (higher) exposure / Towards null / Away from null / Insufficient information available
Is the risk of bias (due to selection of participants into the study) sufficiently high, in the context of its likely direction and the magnitude of the estimated exposure effect, to threaten conclusions about whether the exposure has an important effect on the outcome?	Yes / No / Cannot tell

Y = Yes; PY = Probably yes; PN = Probably no; N = No; SN = Strong no; WN = Weak no; NA = Not applicable; NI = No information

Domain 4: Risk of bias due to post-exposure interventions

Signalling questions	Response options
4.1 Were there post-exposure interventions that were influenced by prior exposure during the follow-up period?	Y / PY / PN / N / NI
4.2 **If Y/PY to 4.1**: Is it likely that the analysis corrected for the effect of post-exposure interventions that were influenced by prior exposure?	NA / Y / PY / PN / N / NI
Risk of bias (due post-exposure interventions) in the estimated effect of exposure on the outcome	Low risk / Some concerns / High risk / Very high risk
What is the predicted direction of bias due to confounding?	Towards benefit of (higher) exposure / Towards harm of (higher) exposure / Towards null / Away from null / Insufficient information available
Is the risk of bias (due post-exposure interventions) sufficiently high, in the context of its likely direction and the magnitude of the estimated exposure effect, to threaten conclusions about whether the exposure has an important effect on the outcome?	Yes / No / Cannot tell

Y = Yes; PY = Probably yes; PN = Probably no; N = No; NA = Not applicable; NI = No information

Domain 5: Risk of bias due to missing data

Signalling questions	Response options
5.1 Were complete data on exposure status available for all, or nearly all, participants?	Y / PY / PN / N / NI
5.2 Were complete data on the outcome available for all, or nearly all, participants?	Y / PY / PN / N / NI
5.3 Were complete data on confounding variables available for all, or nearly all, participants?	Y / PY / PN / N / NI
5.4 **If N/PN/NI to 5.1, 5.2 or 5.3**: Is the result based on a complete case analysis?	NA / Y / PY / PN / N / NI

Signalling questions	Response options
5.5 **If Y/PY/NI**: Was exclusion from the analysis because of missing data (in exposure, confounders or the outcome) likely to be related to the true value of the outcome?	NA / SY (Yes, strongly related) / WY (Yes, but not strongly related) / PN / N / NI
5.6 **If N/PN to 5.5**: Were all or most predictors of missingness (in exposure, confounders or the outcome) included in the analysis model?	NA / SY (Yes, for sure) / WY (Yes, mostly or probably) / PN / N / NI
5.7 **If N/PN to 5.4**: Was the analysis based on imputing missing values?	NA / Y / PY / PN / N
5.8 **If Y/PY to 5.7**: Was imputation performed appropriately?	NA / Y / PY / WN (no, but not leading to substantial bias) / SN (no, such that bias would not be substantially reduced) / NI
5.9 **If N/PN to 5.7**: Was an appropriate alternative method used to correct for bias due to missing data?	NA / Y / PY / WN (no, but not leading to substantial bias) / SN (no, such that bias would not be substantially reduced) / NI
5.10 **If PN/N/NI to 5.1, 5.2 or 5.3**: Is there evidence that the result was not biased by missing data?	NA / Y / PY / PN / N
Risk of bias (due to missing data) in the estimated effect of exposure on the outcome	Low risk / Some concerns / High risk / Very high risk
What is the predicted direction of bias due to missing data?	Towards benefit of (higher) exposure / Towards harm of (higher) exposure / Towards null / Away from null / Insufficient information available
Is the risk of bias (due to missing data) sufficiently high, in the context of its likely direction and the magnitude of the estimated exposure effect, to threaten conclusions about whether the exposure has an important effect on the outcome?	Yes / No / Cannot tell

Y = Yes; PY = Probably yes; PN = Probably no; N = No; SY = Strong yes; WY = Weak yes; NA = Not applicable; NI = No information

Domain 6: Risk of bias arising from measurement of the outcome

Signalling questions	Response options
6.1 Could measurement or ascertainment of the outcome have differed between exposure groups or levels of exposure?	Y / PY / PN / N / NI
6.2 Were outcome assessors aware of study participants' exposure history?	Y / PY / PN / N / NI
6.3 **If Y/PY/NI to 6.2**: Could assessment of the outcome have been influenced by knowledge of participants' exposure history?	NA / SY (yes, to a large extent) / WY (yes, to a small extent) / PN / N / NI
Risk of bias (arising from measurement of outcomes) in the estimated effect of exposure on the outcome	Low risk / Some concerns / High risk / Very high risk
What is the predicted direction of bias arising from measurement of outcomes?	Towards benefit of (higher) exposure / Towards harm of (higher) exposure / Towards null / Away from null / Insufficient information available
Is the risk of bias (arising from measurement of outcomes) sufficiently high, in the context of its likely direction and the magnitude of the estimated exposure effect, to threaten conclusions about whether the exposure has an important effect on the outcome?	Yes / No / Cannot tell

Y = Yes; PY = Probably yes; PN = Probably no; N = No; SY = Strong yes; WY = Weak yes; NA = Not applicable; NI = No information

Domain 7: Risk of bias in selection of the reported result

Signalling questions	Response options
7.1 Was the result reported in accordance with an available, pre-determined analysis plan?	Y / PY / PN / N / NI
7.2 **If N/PN/NI to 7.1**: Is the reported effect estimate likely to be selected, based on desirability of the magnitude (or statistical significance) of the estimated effect of exposure on outcome, from multiple *exposure measurements* within the exposure domain?	NA / Y / PY / PN / N / NI

Signalling questions	Response options
7.3 Is the reported effect estimate likely to be selected, based on desirability of the magnitude (or statistical significance) of the estimated effect of exposure on outcome, from multiple *outcome measurements* within the outcome domain?	Y / PY / PN / N / NI
7.4 Is the reported effect estimate likely to be selected, based on desirability of the magnitude (or statistical significance) of the estimated effect of exposure on outcome, from multiple *analyses* of the exposure-outcome relationship?	Y / PY / PN / N / NI
7.5 Is the reported effect estimate likely to be selected, based on the basis of desirability of the results (e.g. statistical significance), from different *subgroups*?	Y / PY / PN / N / NI
Risk of bias (due to selection of the reported result) in the estimated effect of exposure on the outcome	Low risk / Some concerns / High risk / Very high risk
What is the predicted direction of bias due to selection of the reported result?	Towards benefit of (higher) exposure / Towards harm of (higher) exposure / Towards null / Away from null / Insufficient information available
Is the risk of bias (due to selection of the reported result) sufficiently high, in the context of its likely direction and the magnitude of the estimated exposure effect, to threaten conclusions about whether the exposure has an important effect on the outcome?	Yes / No / Cannot tell

Y = Yes; PY = Probably yes; PN = Probably no; N = No; NA = Not applicable; NI = No information
