# Supplementary material for: Obstetric, foetal and neonatal outcomes in adolescent pregnancy in sub-Saharan Africa: Systematic review and meta-analysis protocol
Source: PLoS One. 2025 May 9;20(5):e0323099. doi: 10.1371/journal.pone.0323099 (PMC12063885; doi:10.1371/journal.pone.0323099)
Supplement: S2 Table — (PDF) [file pone.0323099.s003.pdf]

DATA EXTRACTION FORM

Characteristics of the included studies			
Variable	Description	Location in text	Notes
Study ID*			
Country where study was conducted			
Year study was conducted			
Study design type			
Sample size			
Setting (urban/rural)			
Community/ health facility			
Age			
Parity			
Gravida			
Screening/diagnostic criteria/tool			
Cut-offs of screening/ diagnostic criteria/tool			
Period of screening			
Type of antenatal clinic			
Outcomes assessed			
**Results**			
**Binary/dichotomous outcome (non-comparative)**			
No. of events (n)	Sample size (N)		

**Binary/dichotomous outcome (comparative**					
No. of events (n)	Sample size (N)	No. of events (n)	Sample size (N)		

**Continuous outcome (non-comparative)**				
Mean	SD	Sample size (N)		

**Continuous outcome (comparative)**							
Mean	SD	Sample size (N)	Mean	SD	Sample size (N)		
